# A review of child stunting determinants in Indonesia

**DOI:** 10.1111/mcn.12617

**Published:** 2018-05-17

**Authors:** Ty Beal, Alison Tumilowicz, Aang Sutrisna, Doddy Izwardy, Lynnette M. Neufeld

**Affiliations:** ^1^ Department of Environmental Science and Policy, Program in International and Community Nutrition University of California, Davis Davis California USA; ^2^ Global Alliance for Improved Nutrition (GAIN) Geneva Switzerland; ^3^ Consultant for Global Alliance for Improved Nutrition (GAIN) Jakarta Indonesia; ^4^ Direktorat Gizi Masyarakat‐Kementerian Kesehatan RI Jakarta Indonesia

**Keywords:** child stunting, conceptual framework, determinants, height for age, Indonesia, linear growth

## Abstract

Child stunting reduction is the first of 6 goals in the Global Nutrition Targets for 2025 and a key indicator in the second Sustainable Development Goal of Zero Hunger. The prevalence of child stunting in Indonesia has remained high over the past decade, and at the national level is approximately 37%. It is unclear whether current approaches to reduce child stunting align with the scientific evidence in Indonesia. We use the World Health Organization conceptual framework on child stunting to review the available literature and identify what has been studied and can be concluded about the determinants of child stunting in Indonesia and where data gaps remain. Consistent evidence suggests nonexclusive breastfeeding for the first 6 months, low household socio‐economic status, premature birth, short birth length, and low maternal height and education are particularly important child stunting determinants in Indonesia. Children from households with both unimproved latrines and untreated drinking water are also at increased risk. Community and societal factors—particularly, poor access to health care and living in rural areas—have been repeatedly associated with child stunting. Published studies are lacking on how education; society and culture; agriculture and food systems; and water, sanitation, and the environment contribute to child stunting. This comprehensive synthesis of the available evidence on child stunting determinants in Indonesia outlines who are the most vulnerable to stunting, which interventions have been most successful, and what new research is needed to fill knowledge gaps.

**Key Messages**

- Child stunting is associated with the following determinants in Indonesia: male sex, premature birth, short birth length, nonexclusive breastfeeding for the first 6 months, short maternal height, low maternal education, low household socio‐economic status, living in a household with unimproved latrines and untreated drinking water, poor access to healthcare, and living in rural areas.
- Evidence is lacking for low education; society and culture; agriculture and food systems; and water, sanitation, and the environment contribute to child stunting.
- Evidence gaps and inherent limitations of the World Health Organization conceptual framework prohibited understanding of the causal pathways between individual stunting determinants.

## 1 INTRODUCTION

Under‐five child stunting represents poor linear growth during a critical period and is diagnosed as a height for age less than −2 standard deviations from the World Health Organization (WHO) child growth standards median (WHO, 2006). The consequences of child stunting are both immediate and long term and include increased morbidity and mortality, poor child development and learning capacity, increased risk of infections and noncommunicable diseases in adulthood, and reduced productivity and economic capability (Stewart, Iannotti, Dewey, Michaelsen, & Onyango, 2013). Child stunting reduction is the first of six goals in the Global Nutrition Targets for 2025 (WHO, 2012) and a key indicator in the second Sustainable Development Goal of Zero Hunger (United Nations, Department of Economic and Social Affairs, 2016).

**Figure 1:**
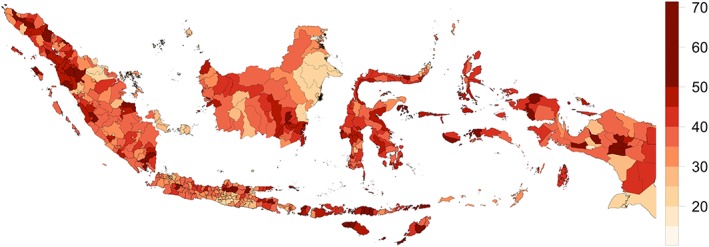
Prevalence of stunting (%) in children 0–59 months by district in 2013. Source: Indonesia basic Health Research survey (Lembaga Penerbitan Balitbangkes Kementerian Kesehatan Republik Indonesia, 2013)

**Figure 2:**
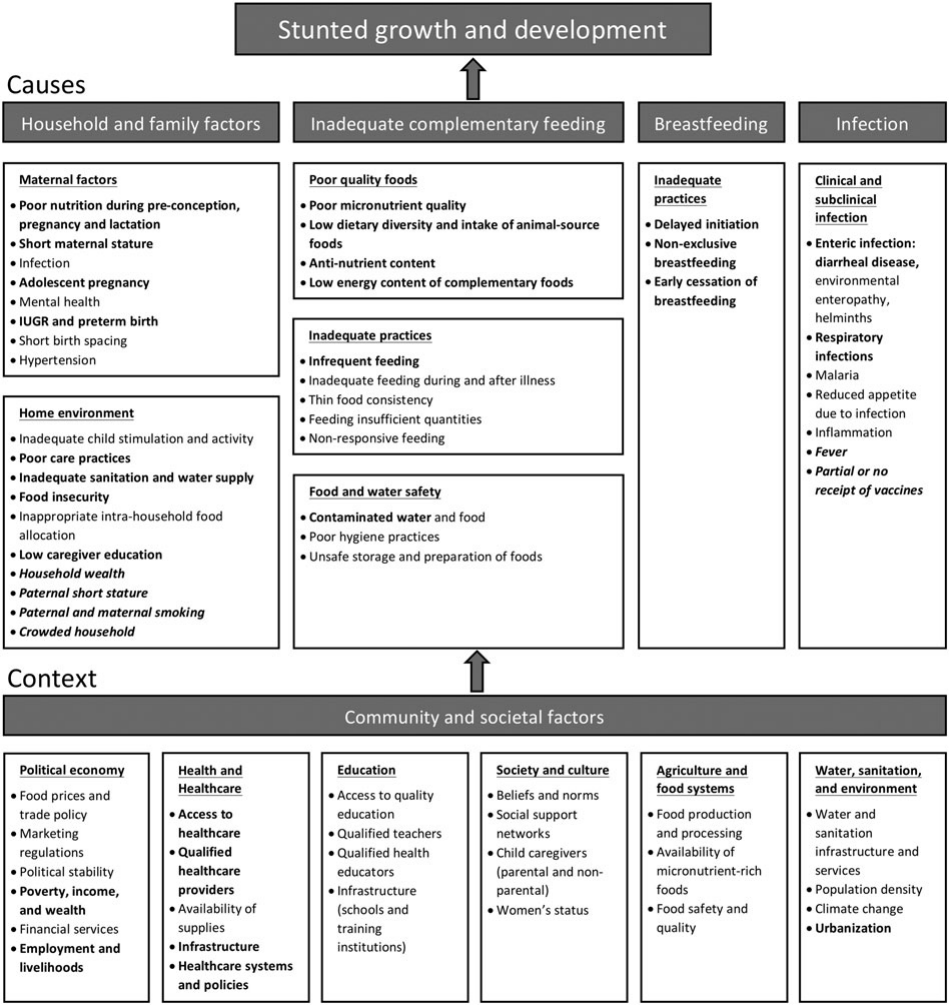
The World Health Organization conceptual framework on childhood stunting: Proximate causes and contextual determinants. Bold text represents determinants that have been addressed in the literature. Normal styled text represents determinants not addressed in the literature. Italicized text represents determinants that were not explicitly stated in the framework but identified in the literature. Modified from Stewart et al., 2013

Over the past decade in Indonesia, there has been little change in the national prevalence of child stunting, which is approximately 37% (National Institute of Research and Development (NHRD), Ministry of Health (MOH), 2013; NHRD, MOH, 2009). There are large disparities subnationally (Figure 1), ranging by province from 26% in Riau Islands to 52% in East Nusa Tenggara (NHRD, MOH, 2013). This indicates the variation in the population's exposure to determinants of child stunting and the need to target and tailor interventions to the most vulnerable. There are numerous potential causes of stunting in Indonesia, including proximate factors such as maternal nutritional status, breastfeeding practices, complementary feeding practices, and exposure to infection as well as related distal determinants such as education, food systems, health care, and water and sanitation infrastructure and services. The purpose of this article is to review the recent literature to determine what has been studied and can be concluded about the determinants of child stunting in Indonesia. We use the WHO child stunting framework (Stewart et al., 2013) to organize studies with an outcome of under‐five child stunting or linear growth into the appropriate determinant categories and identify knowledge gaps (Figure 2).

## 2 METHODS

The WHO framework categorizes the proximate causes of child stunting under these broad elements (and subelements): household and family factors (maternal factors and home environment), inadequate complementary feeding (poor quality foods, inadequate practices, and food and water safety), breastfeeding (inadequate practices), and infection (clinical and subclinical infection). It categorizes corresponding contextual factors under the broad element, community and societal factors, with the following subelements: political economy; health and health care; education; society, and culture; agriculture and food systems; and water, sanitation, and environment. Because the causes and contextual factors of the framework are based on global data, we conducted a literature review to identify determinants within the subelements that have been studied in Indonesia. Determinants in the literature that were not specifically listed in the framework were added under the most relevant subelement. We present the results in narrative summary, commonly used in systematic reviews.

**Figure 3:**
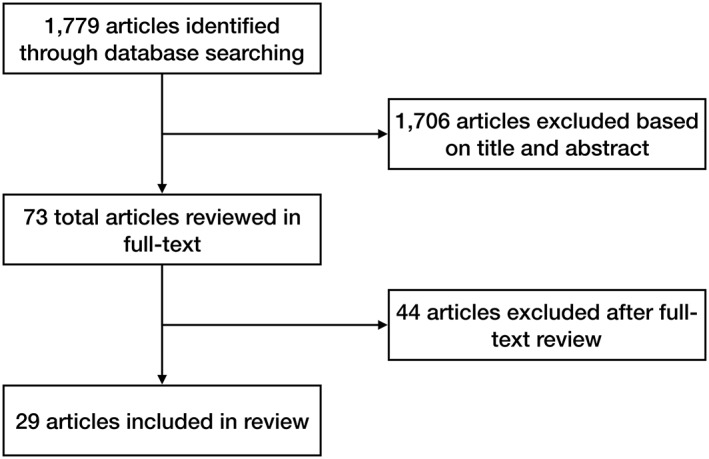
Flow diagram of database search process

To identify child stunting determinants in Indonesia, we conducted keyword searches in PubMed, PubMed Central (PMC), and Web of Science. For PubMed and PMC, we used the following MeSH terms: (“growth disorders”[MeSH Terms]) OR (“growth”[All Fields]) AND (“disorders”[All Fields]) OR (“growth disorders”[All Fields]) OR (“stunting”[All Fields]) AND

(“Indonesia”[MeSH Terms]) OR (“Indonesia”[All Fields]). For Web of Science, we used the keywords “stunting” and “Indonesia.” We limited our search to materials published in or after the year 2000 to ensure relevancy to the current socio‐economic and political conditions. We obtained 86 results from PubMed, 1,624 from PMC, and 69 from Web of Science and selected 29 studies after applying the following inclusion/exclusion criteria (Figure 3):

- Study site: Studies conducted in Indonesia at any level and studies in multiple countries where Indonesia was included—except for global analyses and studies where the primary focus was not relevant to Indonesia.
- Design: Randomized and non‐randomized controlled trials (RCTs) and observational studies.
- Outcome: Stunting or linear growth in children at any age between 0 and 59 months.
- Relevance: Studies published in English that addressed any cause or contextual factor identified in the WHO framework.

To represent the strength of associations between determinants and stunting, we report the relative risk (RR), adjusted odds ratio (AOR), or unadjusted odds ratio (UOR), in descending preferential order, respectively. RR is only available in cohort/controlled study designs and is a preferred metric. The observational studies we included adjusted for different confounding variables depending on the available data and statistical method used by the researchers in the multivariate analysis. We report difference of means and/or change in linear growth when applicable. Statistical associations reported are significant to at least a *p* value less than or equal to 0.05. Ninety‐five percent confidence intervals (CIs) are reported when available. All studies used the WHO Child Growth Standards (2006) except the following that used the National Center for Health Statistics (NCHS) reference population: Barber & Gertler, 2009; Bardosono, Sastroamidjojo, & Lukito, 2007; Berger, de Pee, Bloem, Halati, & Semba, 2007; Best et al., 2008; Fahmida, Rumawas, Utomo, Patmonodewo, & Schultink, 2007; Paknawin‐Mock, Jarvis, Jahari, Husaini, & Pollitt, 2000; Semba, de Pee, et al., 2007; Semba, Kalm et al., 2007.

## 3 RESULTS

### 3.1 Household and family factors

Under this element, the WHO framework includes the subelements maternal factors and home environment. There are eight identified maternal factors: poor nutrition during preconception, pregnancy, and lactation; short maternal stature; infection; adolescent pregnancy; mental health; intrauterine growth restriction (IUGR) and preterm birth; short birth spacing; and hypertension. Of these, poor nutrition during preconception, pregnancy, and lactation; short maternal stature; IUGR and preterm birth; and adolescent pregnancy have been demonstrated to be associated with child stunting in Indonesia.

Only two studies in Indonesia found a modest association between maternal underweight and child stunting (Rachmi, Agho, Li, & Baur, 2016b; Sari et al., 2010). However, several studies in Indonesia have found moderate to strong associations between short maternal stature and child stunting. A cross‐sectional analysis of the Indonesia Nutrition Surveillance System (NSS; 2000–2003), which includes nine rural provinces, found that households with mothers <145 cm tall were associated with an AOR of 2.32 (95% CI [2.25, 2.40]) of maternal and child double burden—defined as a household having a stunted child (6–59 months) and overweight mother—and mothers between 145.0 and 149.9 cm an AOR of 1.63 (95% CI [1.59, 1.68]) when compared with mothers ≥150 cm (Oddo et al., 2012). Semba, de Pee, Sun, et al. (2008) also analysed data from the NSS (2000–2003) and found that higher maternal height was associated with reduced stunting in children 0–59 months (AOR per cm 0.917, 95% CI [0.915, 0.919]), whereas Semba et al. (2011), using the same data, found an association between higher maternal height and reduced stunting in children 6–59 months in rural communities (UOR per cm 0.902, 95% CI [0.900, 0.904]) and urban poor communities (UOR per cm 0.898, 95% CI [0.894, 0.901]). Rachmi et al. (2016b) conducted a secondary analysis of the repeated cross‐sectional Indonesian Family Life Survey (IFLS; 1993, 1997, 2000, and 2007), which includes 13 provinces, and found an AOR of stunting in children 24–59 months of 2.21 (95% CI [1.76, 2.78]) in mothers with a height‐for‐age *Z*‐score (HAZ) <−2 of the WHO Standard Growth Reference for 19‐year‐olds versus mothers of normal height. Finally, A longitudinal RCT with data collected in nine rural villages in Indonesia found that higher maternal height modestly increased length and HAZ in infants 0–12 months (Schmidt et al., 2002).

Three cross‐sectional studies showed a moderate association between younger maternal age and child stunting (Best et al., 2008; Semba et al., 2011; Semba, Kalm, et al., 2007). In these studies, the odds of women ≤24 years having a stunted child were between 1.09 and 1.23 greater than women ≥33 years. Sari et al. (2010) found the opposite association but did not report the strength of the relationship. Results from Oddo et al. (2012) suggest maternal and child double burden is more likely to occur in older women than in younger women, but this is likely due to greater body mass index in older women, not necessarily a greater prevalence of child stunting.

IUGR and preterm birth have been strongly associated with child stunting in Indonesia. In a secondary analysis of data collected between 1995 and 1999 in an RCT in rural Indonesia, premature birth was associated with an RR of 7.11 (95% CI [2.07, 24.48]) of stunting in children 24 months (Prawirohartono, Nurdiati, & Hakimi, 2016). Rachmi et al. (2016b) found that children 24–59 months were less likely to be stunted if at birth they weighed between 2.5 and 3.9 kg (AOR 0.62, 95% CI [0.39, 0.98]) or ≥4 kg (AOR 0.49, 95% CI [0.28, 0.87]), compared with children <2.5 kg in the IFLS. Schmidt et al. (2002) demonstrated that neonatal weight, and particularly neonatal length, was the strongest negative predictors of HAZ and positive predictors of linear growth in infants 0–12 months. Lastly, Semba, de Pee, Sun, et al. (2008) found a decreased risk of stunting in children 0–59 months in the NSS with greater birth weight (AOR per 100 g 0.935, 95% CI [0.933, 0.937]).

The subelement home environment includes inadequate child stimulation and activity, poor care practices, inadequate sanitation and water supply, food insecurity, inappropriate intrahousehold food allocation, and low caregiver education. Studies in Indonesia have found child stunting to be associated with poor care practices, inadequate sanitation and water supply, food insecurity, and low caregiver education. Additional determinants not specifically listed under home environment were found to be associated with child stunting in the literature in Indonesia: indicators of household wealth, paternal and maternal smoking, paternal short stature, and crowded households.

Only one cross‐sectional study reported an association between poor childcare practices and stunting in urban poor children 6–59 months, but it did not reveal the strength of the relationship (Bardosono et al., 2007). The same study also found an association between poor household environmental sanitation (inappropriate latrine facilities) and stunting in rural children 6–59 months (Bardosono et al., 2007). Similarly, Semba et al. (2011) observed that children 6–59 months in households with an improved latrine were less likely to be stunted in rural communities (UOR 0.81, 95% CI [0.79, 0.84]) and urban slums (UOR 0.85, 95% CI [0.81, 0.89]) than households with an unimproved latrine. In a recent study, purchase of inexpensive drinking water—which was assumed to be untreated—was associated with increased odds of stunting in children 0–59 months in urban slums (UOR 1.32, 95% CI [1.20, 1.45]; Semba et al., 2009). Additionally, Torlesse, Cronin, Sebayang, and Nandy (2016) analysed a cross‐sectional survey and demonstrated that children 0–23 months living in a household with untreated drinking water had much higher odds of stunting if the household also used an unimproved latrine (AOR 3.47, 95% CI [1.73, 7.28]). Food insecurity was associated with child stunting in one cross‐sectional study, which found lower odds of stunting (AOR 0.70, 95% CI [0.50, 0.99]) in children 0–23 months in households that consumed more than two meals a day (Ramli et al., 2009).

Low caregiver education, especially maternal education, was strongly associated with child stunting in numerous studies. Bardosono et al. (2007) also observed that inappropriate maternal nutritional knowledge and low paternal education were related to stunting in urban poor children 6–59 months between 1999 and 2001—immediately following the economic crisis in 1999. Four studies found an association between maternal education and child stunting but did not report or include paternal education in their analyses (Berger et al., 2007; Fernald, Kariger, Hidrobo, & Gertler, 2012; Oddo et al., 2012; Schmidt et al., 2002). Three studies reported an association between both paternal and maternal education and child stunting but did not specify which association was stronger (Sari et al., 2010; Semba et al., 2011; Semba, Kalm, et al., 2007). Three studies found an association between paternal education and child stunting but a stronger association between maternal education and child stunting (Best et al., 2008; Rachmi et al., 2016b; Semba, de Pee, Sun, et al., 2008). In general, the odds of child stunting were higher the lower the parental education level, although not unanimously, and the odds of stunting were usually about twice as high for children of parents with the lowest education compared with the highest.

Unsurprisingly, insufficient purchasing power (Bardosono et al., 2007) and other household wealth indicators were strongly associated with child stunting in several cross‐sectional studies throughout Indonesia (Best et al., 2008; Fernald et al., 2012; Ramli et al., 2009; Sari et al., 2010; Semba et al., 2011; Semba, Kalm, et al., 2007; Torlesse et al., 2016). For example, Ramli et al. (2009) found that households with unemployed fathers were associated with a strong increase in odds of severe stunting in children 0–59 months (AOR 2.04, 95% CI [1.17, 3.53]). Moreover, in a more recent analysis, children 0–23 months from households in the lowest wealth quintile compared with those in the highest had an AOR of stunting of 2.30 (95% CI [1.43, 3.68]; Torlesse et al., 2016). Other examples of household expenditure on nutrient‐rich or nutrient‐poor foods—which are more relevant to the category complementary foods—are discussed in further detail under Section 3.2.

Rachmi et al. (2016b) found a strong association between paternal short stature with stunting in children 24–59 months (AOR 1.91, 95% CI [1.51, 2.41]). Additionally, three studies demonstrated a moderate association between crowded households and child stunting (Oddo et al., 2012; Ramli et al., 2009; Semba et al., 2011), whereas many others showed a negligible association (Best et al., 2008; Sari et al., 2010; Semba, Kalm, et al., 2007; Semba, de Pee, Sun et al., 2008). Furthermore, paternal and maternal smoking were modestly associated with stunting in children 0–59 months in only rural areas in one study (Best et al., 2008) and in urban slums and rural areas in another (Semba, Kalm, et al., 2007)—AORs were between 1.10 and 1.17 depending on the model used. Similarly, solely paternal smoking was modestly associated with stunting in both urban poor and rural children 0–59 months in one study (Sari et al., 2010) and only rural children 6–59 months in another (UOR 1.08, 95% CI [1.05, 1.11]; Semba et al., 2011).

Maternal factors not assessed for association with child stunting or linear growth in the literature in Indonesia include infection, mental health, short birth spacing, and hypertension. Home environment determinants not assessed for association with child stunting or linear growth include inadequate child stimulation and activity and inappropriate intrahousehold food allocation.

### 3.2 Inadequate complementary feeding

This element includes poor quality foods, inadequate feeding practices, and food and water safety. The subelement poor‐quality foods includes poor micronutrient quality, low dietary diversity and intake of animal‐source foods, antinutrient content, and low energy content of complementary foods. The subelement inadequate feeding practices includes infrequent feeding, inadequate feeding during and after illness, thin food consistency, feeding insufficient quantities, and nonresponsive feeding. The subelement food and water safety includes contaminated food and water, poor hygiene practices, and unsafe storage and preparation of foods. Research on complementary feeding in Indonesia has focused almost exclusively on poor quality foods (including supplementation and fortification interventions), except one study on contaminated water and one study that peripherally addressed infrequent feeding. Although the impact of probiotics on linear growth is not specifically addressed in the WHO framework, Agustina et al. (2013) found that the probiotic *L* *actobacillus* *reuteri* DSM 17938 modestly increased height velocity compared with the control (0.03 cm/month, 95% CI [0.01, 0.05]) in children 1–6 years living in poor urban communities in Jakarta, Indonesia.

Several studies addressed the micronutrient quality of complementary foods in some way, although most did not directly assess dietary intake of complementary foods. Sari et al. (2010) found that households in the highest quintile of animal‐source food expenditure were associated with a decreased odds of stunting in urban poor children (AOR 0.87, 95% CI [0.85, 0.90]) and rural children (AOR 0.78, 95% CI [0.74, 0.81]) 0–59 months, compared with households in the lowest quintile (Sari et al., 2010). Households in the highest quintile of plant‐source food expenditure were associated with a decreased odds of stunting in rural children 0–59 months (AOR 0.86, 95% [0.84, 0.88]) but not urban poor children, compared with households in the lowest quintile (Sari et al., 2010). Additionally, children 0–59 months from households in the highest quintile of grain food expenditure in rural areas had an AOR of stunting of 1.21 (95% CI [1.18, 1.25]) and in urban slums an AOR of stunting of 1.09 (95% CI [1.09, 1.13]), compared with households in the lowest quintile (Sari et al., 2010). Similarly, Semba et al. (2011) reported decreased odds of stunting with higher household animal‐source food expenditure in rural children (UOR 0.87, 95% CI [0.82, 0.92]) and urban poor children (UOR 0.78, 95% CI [0.72, 0.85]) and decreased odds of stunting with higher household plant‐source food expenditure in rural children (UOR 0.79, 95% CI [0.74, 0.84]) and urban poor children (UOR 0.86, 95% CI [0.79, 0.94]) 6–59 months. In a recent study, households without age‐appropriate feeding—which includes a minimum acceptable diet of adequate diversity and frequency—were associated with increased odds of stunting in children 0–23 months (UOR 1.39, 95% CI [1.09, 1.77]; Torlesse et al., 2016).

Semba et al. (2011) found that intake of multiple micronutrient (MMN)‐fortified milk was associated with decreased odds of stunting in children 6–59 months in rural areas (UOR 0.87, 95% CI [0.85, 0.90]) and urban areas (UOR 0.80, 95% CI [0.76, 0.85]), whereas intake of MMN‐fortified noodles was associated with only modest decreased odds of stunting in rural areas (UOR 0.95, 95% CI [0.91, 0.99]). A recent non‐RCT in rural Indonesia showed that consumption of small‐quantity lipid‐based nutrient supplements (SQ‐LNS)—which provide micronutrients and macronutrients—over 6 months considerably reduced stunting incidence (RR 0.35) in infants 6–12 months compared with the control group (Muslihah, Khomsan, Briawan, & Riyadi, 2016). Aitchison, Durnin, Beckett, and Pollitt (2000) conducted a RCT and found that a supplement with energy (~280 kcal) and iron (12 mg) only modestly increased length in children 12 months and 18 months of age after 6 months of intervention. An analysis of the supplementary feeding programme that took place after the 1997–1998 financial crisis found that children 12–24 months who were involved in the programme for at least 12 months during 2 years experienced a 7% decline in stunting and a 15% decline in severe stunting compared with the control group (Giles & Satriawan, 2015). Lastly, consumption of fruit and biscuits modestly increased length and HAZ in infants 0–12 months in a RCT by Schmidt et al. (2002).

An RCT in four sites in Southeast Asia—two of which were in Indonesia—found that zinc supplementation and not iron supplementation given to children 4–6 months for 6 months resulted in an increased HAZ of 0.17 cm only in anaemic infants (Dijkhuizen et al., 2008). Contrastingly, Fahmida et al. (2007) conducted a double‐blind RCT and found that among initially stunted children 3–6 months, 6 months of supplementation with iron+zinc or iron+zinc+vitamin A resulted in increased length of 1 cm compared with placebo and supplementation with zinc alone. High‐dose vitamin A supplementation was associated with increased linear growth in preschool‐aged children in two studies, particularly among those with very low serum retinol (Hadi et al., 2000; Semba et al., 2011). Specifically, an RCT by Hadi et al. (2000) found that children 6–48 months with a serum retinol concentration <35 μmol/L given high‐dose vitamin A supplements every 4 months had a height increase of 0.39 cm/4 months (95% CI [0.24, 0.53]) greater than the placebo group. In a cross‐sectional study, receipt of vitamin A supplementation in the prior 6 months was modestly associated with reduced odds of stunting in rural children 6–59 months (UOR 0.96, 95% CI [0.93, 0.99]; Semba et al., 2011). The same study observed a slightly stronger association between households using iodized salt and child stunting in rural areas (UOR 0.89, 95% CI [0.87, 0.92]) and urban slums (UOR 0.94, 95% CI [0.90, 0.98]; Semba et al., 2011). Finally, Semba, de Pee, Hess, et al. (2008) found that households with adequately iodized salt were significantly associated with a modestly lower stunting prevalence in children 0–59 months—2.1% in urban slums and 5.2% in rural areas.

As stated previously, purchase of inexpensive drinking water was moderately associated with increased odds of stunting in children 0–59 months in urban slums (Semba et al., 2009). Inadequate feeding practices not assessed for association with child stunting or linear growth in Indonesia include inadequate feeding during and after illness, thin food consistency, feeding insufficient quantities, and nonresponsive feeding. Food and water safety determinants not assessed for association with child stunting or linear growth include contaminated food, poor hygiene practices, and unsafe storage and preparation of foods.

### 3.3 Breastfeeding

Under inadequate breastfeeding practices, the WHO framework includes delayed initiation of breastfeeding, nonexclusive breastfeeding, and early cessation of breastfeeding. One study found no association between children 0–23 months who began breastfeeding within 1 hr after birth and reduced stunting (Torlesse et al., 2016). Two recent analyses by Rachmi et al. (2016b); Rachmi, Agho, Li, and Baur (2016a) demonstrated that children weaned before 6 months had much higher odds of stunting (AOR 3.16, 95% CI [1.91, 523] and AOR 2.98, 95% CI [1.20, 7.41]). The same studies also observed that prolonged breastfeeding was associated with a higher prevalence of child stunting, but there is insufficient evidence in this cross‐sectional study to determine a causal relationship and adequately account for confounding factors. As mentioned under inadequate complementary feeding, Torlesse et al. (2016) found a moderate association between age‐appropriate feeding—which also includes exclusive breastfeeding in children 0–5 months—and reduced child stunting (Torlesse et al., 2016).

### 3.4 Infection

Under clinical and subclinical infection, the WHO framework includes enteric infection (diarrheal disease, environmental enteropathy, and helminths), respiratory infections, malaria, reduced appetite due to infection, and inflammation. Of these, only respiratory infections and one type of enteric infection (diarrheal disease) were addressed in the literature and found to be associated with child stunting. However, the literature revealed determinants not specifically listed in the WHO framework—fever and partial or no receipt of vaccines—that were associated with child stunting.

Bardosono et al. (2007) reported that infectious diseases—including diarrheal disease, respiratory infections, and fever—were associated with stunting in children 6–59 months living in urban poor and rural areas. Although they did not specify the magnitude of this relationship, the prevalence of respiratory infections was highest in all study populations, followed by fever and diarrheal disease. Semba et al. (2011) found a moderately strong association between diarrhoea in the past 7 days and stunting in children 6–59 months, particularly in rural areas (UOR 1.30, 95% CI [1.22, 1.37]). Additionally, Semba, de Pee, et al. (2007) reported that children 12–59 months who had complete, partial, or no receipt of vaccines had stunting prevalences of 37%, 47%, and 54%, respectively. The association between vaccine receipt and severe child stunting was even stronger: 10% for complete, 16% for partial, and 22% for no receipt of vaccines (Semba, de Pee, et al., 2007).

### 3.5 Community and societal factors

Community and societal factors are the sole element under contextual determinants of child stunting in the WHO framework. Subelements include political economy, health and health care, education, society and culture, agriculture and food systems, and water, sanitation, and environment. Of these, studies have found child stunting to be associated with many determinants of political economy and health and health care, and one determinant of water, sanitation, and environment. Because we reported household wealth indicators under home environment, we do not restate them here, though they overlap with determinants under political economy (i.e., poverty, income, and wealth; and employment and livelihoods).

Political economy includes food prices and trade policy; marketing regulations; political stability; poverty, income, and wealth; financial services; and employment and livelihoods. Health and health care includes access to health care, qualified health care providers, availability of supplies, infrastructure, and health care systems and policies. Education includes access to quality education, qualified teachers, qualified health educators, and infrastructure (schools and training institutions). Society and culture includes beliefs and norms, social support networks, child caregivers (parental and nonparental), and women's status. Agriculture and food systems includes food production and processing, availability of micronutrient‐rich foods, and food safety and quality. Lastly, water, sanitation, and environment includes water and sanitation infrastructure and services; population density; climate change; urbanization; and natural and manmade disasters.

Studies in Indonesia have addressed all determinants of health and health care except availability of supplies. Unsurprisingly, inadequate access to health care has been associated with child stunting in multiple studies (Anwar, Khomsan, Sukandar, Riyadi, & Mudjajanto, 2010; Bardosono et al., 2007; Torlesse et al., 2016). Bardosono et al. (2007) found an association between access to health services and HAZ, though the path‐model was a poor fit. In another study, mothers who had less than four antenatal care (ANC) visits during pregnancy were more likely to have stunted children 0–23 months (UOR 1.70, 95% CI [1.12, 2.60]) than those with four or more visits (Torlesse et al., 2016). Finally, Anwar et al. (2010) found that boys under 5 years old with low attendance (1–3 times) to Posyandu (Integrated Health and Nutrition Services) had an average HAZ of −1.9 (*SD* 1.7) compared with boys with high attendance (4–6 times; HAZ −1.3, *SD* 1.8).

Two studies demonstrated a relationship between unqualified health care providers (especially the absence of medical doctors [MDs]) and child stunting (Barber & Gertler, 2009; Torlesse et al., 2016). Torlesse et al. (2016) reported the odds of stunting in children 0–23 months were more than double if a doctor or midwife did not provide ANC (UOR 2.07, 95% CI [1.29, 3.33]). Similarly, a simulation of the cross‐sectional 1993 and 1997 IFLSs suggested that increasing the number of MDs from none to one in children 0–23 months would result in a length gain of 0.27 cm (Barber & Gertler, 2009). Smaller increases in length were found when increasing the number of nurses from none to three or more (0.18 cm) and adding a midwife where no MD exists (0.09 cm; Barber & Gertler, 2009). Only Torlesse et al. (2016) found an association between infrastructure and child stunting: The odds of stunting in children 0–23 months were more than twice as high when ANC was not obtained at a health facility (UOR 2.12, 95% CI [1.16, 3.87]), and even higher for severe stunting (AOR 2.58, 95% CI [1.19, 5.58]). Finally, Paknawin‐Mock et al. (2000) used a cross‐sectional ecological‐economic approach and demonstrated a relationship between both childcare services and community vaccination programs and severe stunting in children 6–18 months—childcare services had a relatively stronger impact than community vaccination programs.

Within the subelement water, sanitation, and environment, the only component studied and found to be associated with child stunting was urbanization, with most studies observing that rural areas have a higher prevalence of child stunting than urban areas, even poor urban areas. Rachmi et al. (2016b) estimated that the prevalence of stunting in children 24–59 months was 53.3% (95% CI [51.2, 55.4]) in rural areas compared with 34.9% (95% CI [32.9, 37.0]) in urban areas, with a AOR of stunting of 1.55 (95% CI [1.22, 1.97]) in rural versus urban areas. Sandjaja et al. (2013) analysed a cross‐sectional survey and found a similar difference in the prevalence of stunting in the same age group—rural 47.3% and urban 28.5%. Semba, de Pee, Hess, et al. (2008) found that the odds of stunting in children 0–59 months were moderately higher in rural versus urban settings (AOR 1.136, 95% CI [1.075, 1.202]). One study reported that the odds of stunting were higher in urban areas compared with rural areas in children 0–59 months (UOR 1.33, 95% CI [1.03, 1.71]), but it was a cross‐sectional study conducted only within North Maluku province, and the 95% CIs overlapped for the stunting prevalence estimates (rural 33.4%, 95% CI [28.6, 38.6] and urban 40.0%, 95% CI [37.2, 42.9]; Ramli et al., 2009).

Community and societal factors not assessed for association with child stunting or linear growth in Indonesia include availability of health supplies, water and sanitation infrastructure and services, population density, climate change, food prices and trade policy, marketing regulations, political stability, financial services, and all determinants within the subelements education, society and culture, and agriculture and food systems.

## 4 DISCUSSION

The WHO conceptual framework enabled a thorough review of the literature on child stunting determinants in Indonesia. Our results demonstrate there is strong and consistent evidence from RCTs and observational studies that household and family factors—short maternal stature, premature birth, short birth length, low maternal education, and low household wealth—are important proximate determinants of child stunting in Indonesia. Recently, well‐designed cross‐sectional studies suggest early cessation of breastfeeding, short paternal stature, and households with both untreated drinking water and unimproved latrines may also be strong determinants of child stunting in Indonesia, but more research is needed to confirm these results. Additionally, a recent non‐RCT in rural West Madura Island suggests providing children with SQ‐LNS may considerably reduce child stunting in rural Indonesia. Although SQ‐LNS are a relatively new preventative treatment for child stunting, large‐scale RCTs in Ghana and Burkina Faso have also shown promising results (Adu‐Afarwuah et al., 2007; Hess et al., 2015). However, SQ‐LNS do not improve linear growth in all child populations—as demonstrated in rural Malawi—and other factors such as adherence to the intervention, subclinical infections, environmental enteropathy, or an unbalanced intestinal microbiome may limit the impact of SQ‐LNS (Ashorn et al., 2015). Interventions using MMN supplements (Smuts et al., 2005; Untoro, Karyadi, Wibowo, Erhardt, & Gross, 2005) or solely small‐energy supplements (Aitchison et al., 2000) have not shown an effect on linear growth or child stunting in Indonesia; however, certain individual micronutrients (vitamin A, zinc, and iodine) and combinations of iron+zinc and iron+zinc+vitamin A have (Dijkhuizen et al., 2008; Fahmida et al., 2007; Hadi et al., 2000; Semba et al., 2011; Semba, Pee, Hess, et al., 2008). We also found that community and societal factors have been shown to play an important role in child stunting in Indonesia—particularly access to health care, health infrastructure, and qualified health providers (especially MDs). Figure 2 more comprehensively shows what proximate causes, and contextual factors have been associated with poor linear growth and/or child stunting in Indonesia (bold text).

Wirth et al. (2017) conducted a similar analysis to ours using the WHO framework to assess child stunting determinants in Ethiopia. Child birth size and recent illness, and maternal stature and education were the strongest determinants identified in Ethiopia (Wirth et al., 2017). Our findings in Indonesia of child birth size (especially birth length and premature birth) and maternal stature and education correspond, furthering the evidence that stunting begins in utero (Neufeld, Haas, Grajéda, & Martorell, 2004). This highlights the importance of reaching adolescent girls, because young women who become pregnant while facing undernutrition are at increased risk of poor birth outcomes that can lead to child stunting. Interventions starting at or after birth can only have a limited impact in children who were stunted in utero. Although recent child illness—such as diarrhoea and respiratory infection—was associated with child stunting in Indonesia, the evidence was limited and the strength of the relationship weaker than in Ethiopia.

This study has several strengths. The database search and article selection process involved a thorough assessment of all published scientific studies with an outcome of under‐five child stunting or linear growth in the past 17 years in Indonesia, based on a priori inclusion criteria to limit bias. We reported quantitatively the strength of associations using RRs, odds ratios, and/or difference in means as well as corresponding CIs, while also providing a qualitatively nuanced discussion of the study populations, interventions, variable definitions, and outcomes. To our knowledge, no similar assessment of child stunting determinants in Indonesia has been conducted, and few comprehensive assessments of child stunting determinants have been carried out at the national level in other countries. This information is critical to create effective interventions and policies aimed at reducing child stunting in low‐ and middle‐income countries and to identify priorities for future research.

We identified several factors with significant associations with child stunting in Indonesia that are not specifically listed in the WHO framework: low household wealth, paternal short stature, paternal and maternal smoking, crowded households, fever, and partial or no receipt of vaccines. Household wealth indicators, however, may also be represented under political economy, depending on how they are classified. Additionally, paternal short stature may be strongly correlated with maternal short stature and may not provide any new insight. Likewise, household wealth may be partially represented by food insecurity, although wealth facilitates additional health benefits such as access to health care and medicine. Wirth et al. (2017) specified missing determinants, mostly under home environment, from findings in Ethiopia, but also from studies in other countries. Among others, household wealth and family size were similarly identified in our study as likely important determinants in Indonesia.

We also found substantial evidence that boys were at a much greater risk of stunting than girls in Indonesia, including one longitudinal RCT, but sex‐based biology is not in the WHO framework (Julia, van Weissenbruch, Delemarre‐van de Waal, & Surjono, 2004; Prawirohartono et al., 2016; Rachmi et al., 2016b; Ramli et al., 2009; Sandjaja et al., 2013; Sari et al., 2010; Semba, de Pee, Hess, et al., 2008; Semba et al., 2011; Torlesse et al., 2016). Although boys are generally more susceptible to stunting than girls in developing countries, the mechanism for this is poorly understood (Bork & Diallo, 2017). One possible explanation is a convergence of biological factors, living conditions, and differences in maternal feeding patterns of boys due to gendered cultural perceptions (Tumilowicz, Habicht, Pelto, & Pelletier, 2015).

Although it is impractical to list every possible child stunting indicator in a conceptual framework, it may be worthwhile to consider adding missing determinants to the WHO framework that have been shown to have a consistent and strong relationship with child stunting, especially those that have been found in multiple countries. There is also confusion about how certain determinants are classified, because there is inevitably overlap between subelements, especially between proximate determinants and contextual factors that address the same topics (e.g., “low caregiver education” under household and family factors versus “education” under community and societal factors). For instance, Wirth et al. (2017) suggests household wealth should be an added indicator under home environment, but they do not consider it under political economy.

A primary limitation of this review is that we did not conduct a meta‐analysis. However, because much of the heterogeneity between included studies was qualitative, a narrative review allowed for an in‐depth discussion of the similarities and differences between studies, which included observational and experimental designs. Another limitation is that many of the studies included in the review were cross‐sectional in design and some analysed data from the same surveys. Cross‐sectional studies are unable to account for unknown confounding variables. Therefore, associations between variables in cross‐sectional studies should be interpreted with caution, because a causal relationship cannot be confirmed.

Another limitation is that only about half of the determinants listed in the WHO conceptual framework have been assessed for their relationship with child linear growth or stunting in Indonesia. Many additional determinants have been studied in Indonesia, but an assessment of their measurable impact on child linear growth or stunting is still needed to provide recommendations for intervention. Nevertheless, the WHO framework was based on repeated evidence from studies throughout the developing world, and until gaps in knowledge in Indonesia can be adequately addressed, it is reasonable to assume, from a programmatic perspective, that the identified determinants are likely relevant to varying degrees in Indonesia. Although the WHO conceptual framework was effective for identifying a broad range of stunting determinants in Indonesia from the available literature, it did not allow for an understanding of the causal pathways between individual determinants or provide sufficient insight into which interventions can best address these pathways. Finally, given the diverse geography and culture in Indonesia, child stunting determinants likely vary geographically, and spatial analysis of the strongest determinants would help identify where to focus interventions and how they could be tailored regionally.

## 5 CONCLUSION

The evidence in Indonesia primarily aligns with common proximate causes of child stunting identified in the broader literature: maternal height and education, premature birth and birth length, exclusive breastfeeding for 6 months, and household socio‐economic status. Unsurprisingly, clean drinking water is especially important for households with unimproved latrines. SQ‐LNS have potential to considerably reduce child stunting incidence, particularly in rural Indonesia, likely due to the provision of both micronutrients and macronutrients during the initial critical growth period when complementary foods are first introduced. Several proximate determinants identified in the WHO framework have not been assessed for their impact on child stunting in Indonesia, and studies addressing these knowledge gaps in Indonesia are needed. Community and societal factors are also vital—especially addressing health and health care—but more research is needed to address the pathways between the political economy, education, society and culture, agriculture and food systems, and water, sanitation, and the environment and child stunting, which likely play an important role in Indonesia.

In addition to mothers of short stature and poor education, children born prematurely, and poor households, children from poor urban and especially rural communities are particularly vulnerable to stunting. Boys are far more likely than girls to be stunted throughout Indonesia; the biological factors, living conditions, and differences in maternal feeding patterns that likely converge to cause sex differences in growth should be a high priority for further investigation. Interventions to prevent child stunting should begin before conception to improve nutritional status during adolescence and pregnancy and facilitate adequate gestational growth, and continue at least until the child is 24 months. Spatial analysis of secondary data containing identified child stunting determinants should be conducted to allow interventions to vary geographically according to the local context. At the very least, given the large regional disparity in child stunting prevalence in Indonesia, interventions should target provinces (or preferably regencies or districts) with the highest burden of child stunting.

## CONFLICTS OF INTEREST

The authors declare that they have no conflicts of interest.

## CONTRIBUTIONS

All authors were involved in developing the paper concept. TB analysed data and wrote the first draft of the manuscript. All authors critically reviewed the content and approved the final version submitted for publication.
